# Evaluation of Improved Imaging Properties with Tungsten-Based Parallel-Hole Collimators: A Monte Carlo Study

**DOI:** 10.1055/s-0044-1786165

**Published:** 2024-04-12

**Authors:** Jalil Pirayesh Islamian, Michael Ljungberg

**Affiliations:** 1 Department of Medical Physics, Faculty of Medicine, Tabriz University of Medical Sciences, Tabriz, Iran; 2 Department of Medical Radiation Physics, Lund, Lund University, Lund, Sweden

**Keywords:** collimator, gamma camera, planar imaging, SIMIND, tungsten, spatial resolution, Wolfmet

## Abstract

**Objectives** The purpose of a parallel-hole collimator in a scintillation camera system is to transmit only those photons that have an emission angle close to the direction of the hole. This makes it possible to receive spatial information about the origin of the emission, that is, radioactivity decay. The dimension, shape, and intrahole thickness determine the spatial resolution and, by a tradeoff, sensitivity. The composition of the collimator material also plays an important role in determining a proper collimator. In this study, we compared tungsten alloys as a potential collimator material replacement for the conventional lead antimony material used in most of the current camera systems.

**Materials and Methods** Monte Carlo simulations of a commercial scintillation camera system with low energy high resolution (LEHR), medium-energy (ME), and high-energy (HE) collimators of lead, tungsten, and tungsten-based alloy were simulated for different I-131, Lu-177, I-123, and Tc-99m sources, and a Deluxe rod phantom using the SIMIND Monte Carlo code. Planar images were analyzed regarding spatial resolution, image contrast in a cold source case, and system sensitivity for each collimator configuration. The hole dimensions for the three collimators were those specified in the vendor's datasheet.

**Results** Using Pb, W, and tungsten alloy (Wolfmet) as collimator materials, the full width at half maximum (FWHM) measures for total counts (T) for LEHR with Tc-99m source (6.9, 6.8, and 6.8 mm), for ME with Lu-177 source (11.7, 11.5, and 11.6 mm), and for HE with I-131 (6.2, 13.1, and 13.1 mm) were obtained, and the system sensitivities were calculated as 89.9, 86.1, and 89.8 cps _T_ /MBq with Tc-99m source; 42.7, 17.4, and 20.9 cps _T_ /MBq with Lu-177 source; and 40.1, 69.7, and 77.4 cps _T_ /MBq with I-131 source. The collimators of tungsten and tungsten alloy (97.0% W, 1.5% Fe, 1.5% Ni) provided better spatial resolution and improved image contrast when compared with conventional lead-based collimators. This was due to lower septal penetration.

**Conclusion** The results suggest that development of a new set of ME and HE tungsten and tungsten alloy collimators could improve imaging of I-131, Lu-177, and I-123.

## Introduction

A collimator in scintillation camera is mandatory for creating an image of the in vivo radiopharmaceutical distribution. It usually consists of a large number of parallel holes in a block of high-attenuating material, and the purpose of these holes is allow only those photons traveling parallel to the collimator holes to pass through and interact in the crystal and contribute to the image. The dimensions of the holes (hole diameter and length) and the septum between them together with the collimator material, for a specific photon energy, determine the spatial resolution and the system sensitivity and thus have an impact on the image quality. 1 2 3 4 5 6 7 Lead (Pb), added with a small fraction of antimony (Sb), is the most commonly used collimator material for full-sized clinical scintillation cameras, and the collimators are, based on this material, optimized to imaging photons ranging from 140 to 364 keV. However, several studies have suggested alternative materials, such as tungsten (W), gold (Au), uranium (U), and platinum (Pt), all of which have better attenuating properties for photons with reduced collimator scatter and septal penetration. 2 8 9 10 11 12 For example, the attenuation coefficients, μ, for tungsten at 140.5 and 364 keV are 36.23 and 4.34/cm, respectively. This can be compared with 26.86 and 3.13/cm for Pb. 13 However, the increased cost and the increased weight of such collimators have made these more useful for smaller collimators, such as the pinhole collimator where the actual aperture can be made by the materials mentioned above but the surrounding collimator housing is made by Pb. 14 15 For examples, the new clinical CZT-based multidetector single-photon emission computed tomography (SPECT) systems, including GE StarGuide (GE Healthcare, Inc) and Veriton (Spectrum Dynamics, Inc), are equipped with tungsten parallel-hole collimators. These systems are, however, based on smaller detectors than a full-sized camera and the collimators are optimized for 140 keV. Also, the dedicated cardiac cameras, D-SPECT (Spectrum Dynamics, Inc) and Discovery NM 530 (GE Healthcare, Inc), are both based on tungsten collimators.

A drawback with tungsten is its high melting point and that it cannot be cast like Pb-based collimators and the metal is also difficult to machine. This makes pure tungsten collimators more expensive than Pb collimators. An alternative is using tungsten in alloys (with nickel, iron, and/or copper), which can then be precisely milled or drilled with a diamond drill or machined using electric discharge machining (EDM). However, these techniques are also very expensive, and also complex shapes like strongly tilted pinholes, loftholes, or pinholes with small opening angles cannot be easily produced. 2 However, pure tungsten can be of interest as a collimator material for magnetic resonance (MR) compatible SPECT systems because tungsten alloys can often contain magnetic materials. 16

More recently, there have been new developments in collimator production techniques, which make the fabrication of more complex collimator designs possible. 17 These include direct three-dimensional (3D) printing of metals and “cold casting” of W-based composite materials. This opens new possibilities for more tailored collimator designs that otherwise would be impossible or very expensive to construct by conventional methods such as casting hot lead (micro-cast) or folding lead foils (micro-linear). 17 18 19 20 21 22 W-based collimators with a mass density of 18.56 g/cm ^3^ and a mean deviation of 35 µm were recently reported for a tungsten parallel-hole collimator with a hole size of 0.525 mm, septal thickness of 0.150 mm, and hole length of 25 mm. 15

Könik et al have modeled two multipinhole (MPH) collimator designs with 9- and 16-pinhole apertures in a 2-cm-thick tungsten alloy (tungsten 90% and Cu 10%; mass density: 18.2 g/cm ^3^ ) to study primary, scatter, and penetration characterizations of parallel-hole and pinhole collimators for I-123 SPECT and found that the MPH collimators provide superior count performance in terms of high primary counts, low penetration, and low scatter counts compared with the Pb-based parallel-hole and single pinhole collimators. 23

For full-size cameras, there are today three families of collimators, namely, low-energy (LE) collimators, medium-energy (ME) collimators, and high-energy (HE) collimators. LE collimators can be provided as optimized for low energy high sensitivity (LE-HS), for spatial resolution (LEHR), or as low energy all purpose collimator (LEAP), and all collimators are mainly used for imaging photons with energies of 140.5 keV (Tc-99m) or below. The ME collimators are generally optimized for In-111 (245 keV) and Ga-67 (300 keV). HE collimators are mainly optimized for I-131. A LEHR collimator can be useful for I-123 imaging because the principal photon energy, 159 keV, is close to 140.5 keV, but the decay also includes emission of photon with energies above 400 keV, which can contribute negatively to the image quality. This is also the case for I-131 that emits two HEs (637 and 364 keV) that both make a significant contribution to the counts in the main energy, 364.5 keV. 24

The aim of this study was therefore to study a potential benefit of using W-based metals as a possible collimator material to reduce the problems mentioned earlier. The study was conducted using Monte Carlo simulations to compare images, obtained from W-based material, with conventional Pb-based collimators regarding system spatial resolution, sensitivity, and image contrast. The study was based on planar imaging since the main purpose was to investigate the influence of the collimator material so the result should be translated to SPECT, since reconstructions are based on planar projection images. Since the aim was not to find an optimal collimator design, regarding collimator hole properties (diameters, lengths, and septum thicknesses), the study was based on commercial collimators but with different collimator materials.

## Materials and Methods

The SIMIND Monte Carlo program was used to simulate a Siemens scintillation camera, equipped with a 0.375-inch-thick NaI(Tl) crystal and conventional HE, ME, and LEHR parallel-hole collimators ( Table 1 ). 25 26 The energy resolution was defined to 9.5% at 140 keV and the intrinsic spatial resolution was set at 3.5 mm. A single slab of Pyrex with a thickness of 6 cm was used as a substitute for photomultiplier tubes (PMTs), electronics, and other compartments to include potential contribution due to backscattering of those emitted photons with higher energy at the principal photopeak energy. 27 The energy-dependent cross-sectional values for the collimator materials, shown in Table 1 , were generated from the XCOM database. 13

**Table 1:** Details on the materials used as a parallel-hole collimator

Material	Composition	Mass density (g/cm ^3^ )	Effective atomic number
Pb	Pb (98%) Sb (2%)	11.25 –	81.63 –
W	W (100%)	19.25	74.0
Wolfmet	W (97.0%) Ni (1.5%) Fe (1.5%)	18.5	72.6

The choice of the composition of the W alloy was based on Wolfmet HE-397 metal, 28 and this specific alloy, with properties described in Table 1 , was selected from several types of alloys that have been described in a previous study. 29 The specifications of the collimators used in this work were taken from the vendor's data sheet.

The weights of the W-based collimators presented in Table 2 were estimated by scaling the Pb weight with the fraction of the W and Wolfmet mass density to the Pb mass density, respectively. A small overestimation is expected here since not all parts of a collimator are made by lead.

**Table 2:** Specifications of the hexagonal parallel-hole collimators

	Collimator type		
Specifications	HE	ME	LEHR
No. of holes	8,000	14,000	148,000
Hole diameter (mm)	4.00	2.94	1.11
Hole length (mm)	59.70	40.64	24.05
Septum thickness (mm)	2.00	1.14	0.16
Weight a of Pb (kg)	124.7	63.5	22.1
Weight of W (kg)	211.7	107.8	37.5
Weight of Wolfmet (kg)	200.1	101.9	35.5

a Weight of Pb collimators taken from the vendor's data sheet.

The isotopes I-123 and I-131 were selected for this study since both have decay schemes that include emission of HE photons that are known to cause penetration problems and contribute to degradation of image quality. Lu-177 was selected due to its common use. It has some HE photons in the decay, but these have a relative low abundance. Tc-99m was selected as a common isotope for imaging that do not have emissions in the decay of photons with HE than the principal energy.

Simulations were made for four types of the sources, namely (1) a point source from the lower surface of the collimator; (2) a uniform circular source distribution with an 11-cm radius that included six cold circular areas with radii equal to the spheres in the NEMA IC phantom (30.0, 18.5, 14.5, 11, 8.5, and 5 mm); and (3) a hot source geometry, similar to the cold source, but the sources with a relative activity concentrations of 2, 3, 4, 6, 8, and 12; and (4) a two-dimensional (2D) model of the hot rods of the Plexiglass Deluxe ECT (model ECT/DLX/P) bar phantom in a cold background. All sources were located at a distance of 10 cm from the lower surface of the camera. A main 15% energy window width was set over the photopeak energies of 140.5, 159, 208.4, and 364.5 keV for Tc-99m, I-123, Lu-177, and I-131 isotopes, respectively. A complete decay spectrum was used for each isotope in the simulations. The matrix size was set to 256 × 256 with 1.12-mm pixel size (zoom factor 4) for the point source simulations and 128 × 128 with 2.24-mm pixel size (zoom factor 2) for the other source distributions.

The system's spatial resolution and the sensitivity in the whole field of view (cps/MBq) were determined from the simulated planar images of the point source. A Gaussian fitting function was used to calculate the full width at half maximum (FWHM) of the point spread function (PSF) from two types of images representing (1) the total counts in the images and (2) the counts created only from geometrically collimated photons to determine the impact on septal penetration. The image contrast was calculated for the two largest circles in the uniform activity phantom (cases with cold sources). To calculate the contrast, the average counts, *C̅ _roi_* , were calculated from a circular region of interest (ROI) with radii equal to a fraction of 0.6 of the physical radii of the ROIs to reduce the partial volume effect. A background ROI was positioned in the center of the source with a radius that will not interfere with the other ROIs.

*Contrast* = [1 = *C̅ _roi_* / *C̅ _bkg_* ] × 100%

## Results

**Fig. 1:**
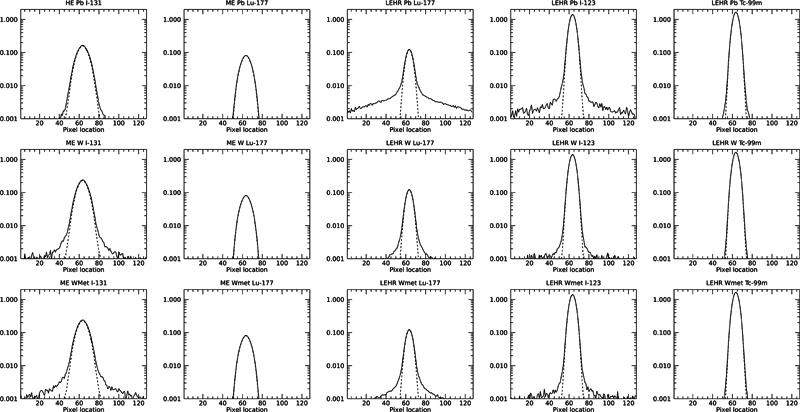
Point spread functions (PSFs) obtained for the three collimators and for I-131, Lu-177, I-123, and Tc-99m isotopes from a point source located 10 cm from the lower surface of the collimator. The *dashed line* represents counts from geometrically collimated photons along the profile and the *solid line* represents all registered counts. ME, medium energy.

PSFs from the events for the isotopes in the simulated images, created by geometrically collimated photons and from all photons, are shown in Fig. 1 for the HE, ME, and LEHR collimators of Pb, W, and Wolfmet. The results of the calculation of the FWHM, the system sensitivity, and the calculated fraction of geometrically collimated counts to the total counts are tabulated in Table 3 . The vendor's specification for the spatial resolution of HE, ME, and LEHR collimators were 13.4 mm (I-131), 12.5 mm (Ga-67), and 7.5 mm (Tc-99m), respectively. When compared with the simulated data of spatial resolution, the FWHM from the simulated Pb collimators showed a good agreement. The vendor's specifications for septal penetration were 1.5, 1.2, and 3.5%, which were generally lower than what we found in our study. This difference can be subjected to how the septal penetration is defined. The definition of septal penetration event in SIMIND is such as when the position of the photon at the exit surface of the collimator is outside the physical boundaries of the current entrance hole plus one septal thickness.

**Table 3:** Results of the spatial resolution for total counts (T), the geometrical collimated counts (G), the system sensitivity in units of cps(T)/MBq and the G/T fraction of the counts for a 10-cm source-to-collimator distance

Radionuclide	Collimator	FWHM(T)	FWHM(G)	Sensitivity	1-G/T
I-131	HE (Pb)	6.2	6.2	40.1	28%
	ME (W)	13.1	12.2	69.7	45%
	ME (Wolfmet)	13.1	12.2	77.4	50%
Lu-177	ME (Pb)	11.7	11.4	11.7	6%
	ME (W)	11.5	11.3	11.3	5%
	ME (Wolfmet)	11.6	11.3	11.4	5%
Lu-177	LEHR (Pb)	7.4	7.2	42.7	84%
	LEHR (W)	7.3	7.1	17.4	61%
	LEHR (Wolfmet)	7.3	7.1	20.9	67%
I-123	LEHR (Pb)	7.0	6.9	111.3	34%
	LEHR (W)	6.9	6.8	91.4	21%
	LEHR (Wolfmet)	6.9	6.8	94.0	23%
Tc-99m	LEHR (Pb)	6.9	6.8	89.9	7%
	LEHR (W)	6.8	6.7	86.1	4%
	LEHR (Wolfmet)	6.8	6.8	86.8	4%

Abbreviations: FWHM, full width at half maximum; HE, high energy; ME, medium energy; ROI, region of interest.

**Fig. 2:**
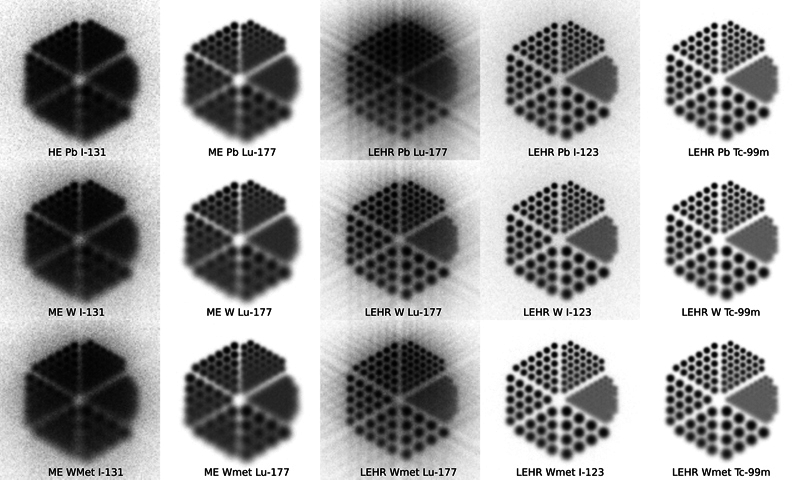
Planar images of the bar sources at 10 cm from the collimator for the three collimators and the I-131, Lu-177, I-123, and Tc-99m isotopes. HE, high energy; ME, medium energy.

When comparing HE Pb with ME W/Wolfmet collimators for I-131 imaging, it can be seen that there is a higher spatial resolution in W-based collimators. The difference in FWHM between W and Wolfmet is very little, but with an increase in penetration for the Wolfmet, as expected due to the lower mass density. However, when viewing the bar images, shown in Fig. 2 , it is hard to see any visible difference. This suggests that an ME W-based collimator could be used to obtain the same image quality as the HE Pb collimator. The sensitivity for the W-based ME collimator is higher and this is not due to septal penetration only since the peaks in the PSF also are higher. Thus, an advantage can be found in both spatial resolution and sensitivity when using ME W-based collimators but at the expense of an increased septal penetration, as can be seen from the tails of the PSF in Fig. 1 .

For Lu-177 imaging, the results showed that there was little advantage of using W-based ME collimators. The PSFs are almost identical in shape and the FWHM values differ very marginally. The penetration problem is thus a small problem, which indicated that ME Pb collimator works fine with Lu-177 and may actually be suboptimal with the 208-keV energy. When imaging Lu-177 with an LEHR W-based collimator and compared to an ME Pb collimator, there was a major improvement in spatial resolution. For LEHR Pb collimators, however, a large fraction of penetration as can be seen, as shown in Table 3 and Fig. 1 . These collimators are therefore not recommended for imaging with 208 keV. 30 However, a potential W-based LEHR collimator significantly reduced the penetration and improved the spatial resolution. The sensitivity is also higher due to the extended number of holes. There are, however, penetration effects that might need to be accounted for, but the improvement in image quality is clearly seen from the bar phantom simulation, shown in Fig. 2 . The difference in sensitivity between W and Wolfmet was marginal.

For I-123 imaging, an LEHR W-based collimator provide a higher spatial resolution and the negative effect of septal penetration can be seen as being small. The sensitivity is slightly lower than that for an LEHR Pb collimator, but the fractions of events from geometrically collimated photon are higher. From Table 4 , we can see that the image contrast for I-123 with W-based collimators is increased. For Tc-99m, there was little advantage with LEHR W collimators in the present configuration. The penetration was small for Pb-based collimators and there was no improvement in spatial resolution and image contrast when using W-based collimators.

**Table 4:** Image contrast for the two circles in Fig. 4 with a radius of 30 and 14.5 mm (the largest and the third largest)

		30-mm ROI			14.5-mm ROI		
Isotope	Collimator	Pb	W	Wolfmet	Pb	W	Wolfmet
I-131 a	HE/ME	83%	73%	59%	76%	64%	59%
Lu-177	ME	100%	100%	98%	97%	97%	98%
	LEHR	51%	70%	52%	36%	58%	52%
I-123	LEHR	77%	84%	81%	73%	82%	81%
Tc-99m	LEHR	99%	100%	99%	98%	100%	99%

Abbreviations: HE, high energy; ME, medium energy; ROI, region of interest.

a ME collimators is used for W-based materials.

**Fig. 3:**
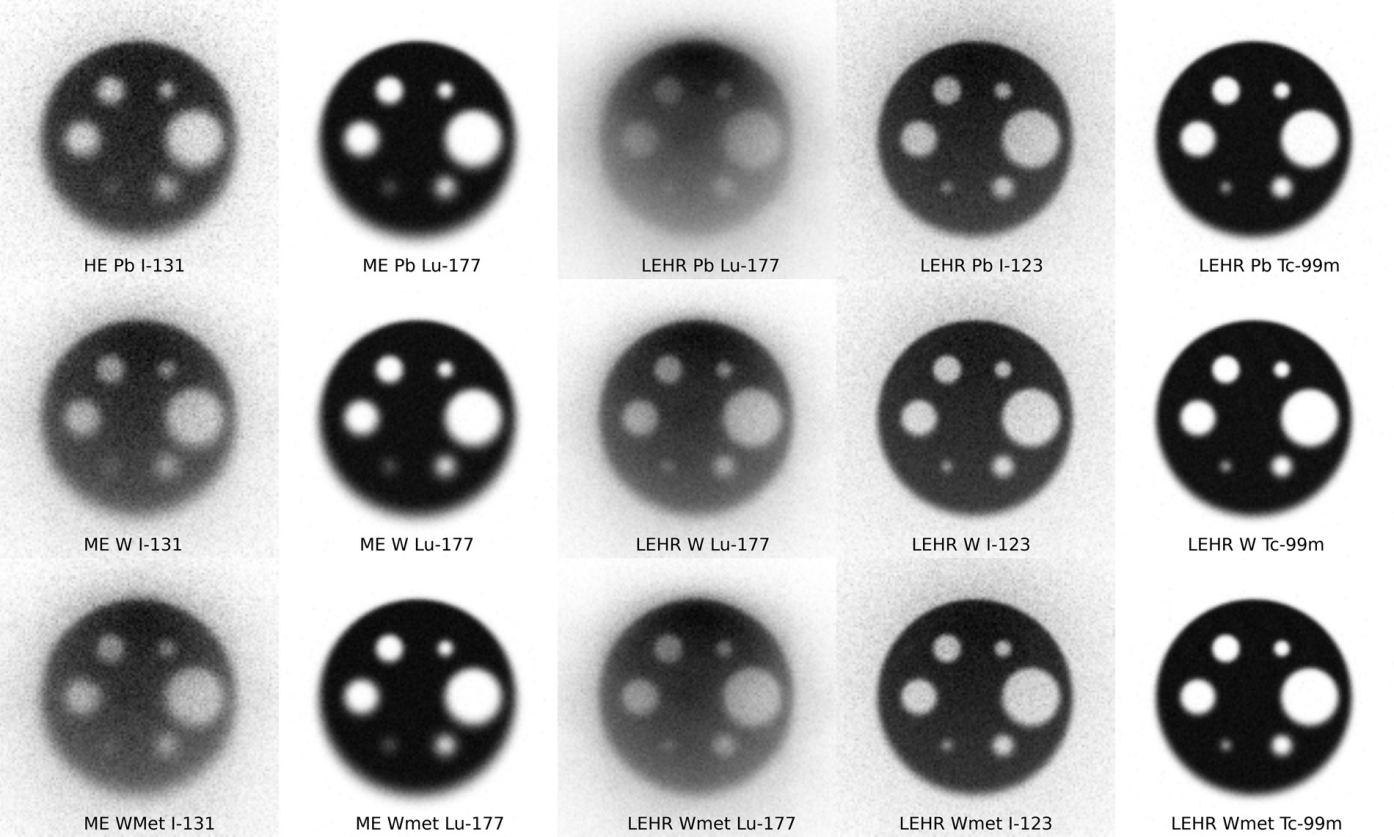
Planar images of uniform distribution with the six cold spheres at 10 cm from the collimator for the three collimators and the I-131, Lu-177, I-123, and Tc-99m isotopes. HE, high energy; ME, medium energy.

**Fig. 4:**
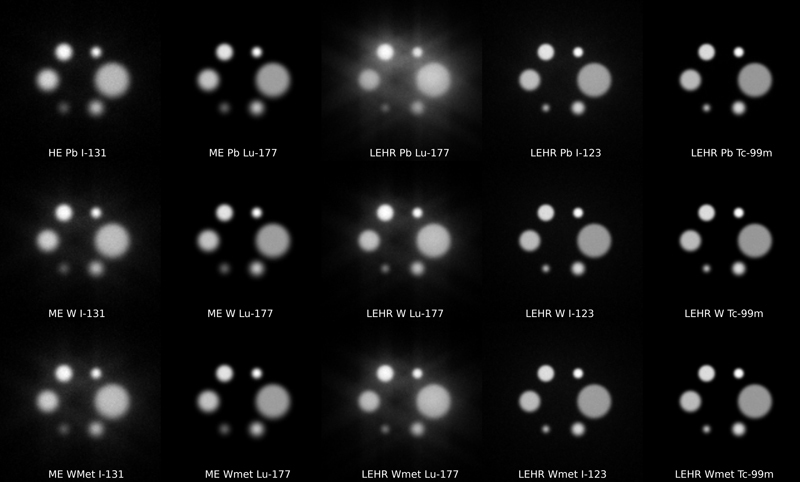
Planar images of the six hot spheres with relative activity concentrations of 2, 3, 4, 6, 8, and 12 for the three collimators and the I-131, Lu-177, I-123, and Tc-99m isotopes. HE, high energy; ME, medium energy.

Fig. 3 shows the cold-cased phantom with six cold circles. The calculated contrast between the ROIs inside the two largest circles is shown in Table 4 . For I-131, the contrast is lower for W-based collimators than that for Pb, indicating the contribution of the surrounding activity to the counts in the ROI due to the septal penetration. Visually the differences are subtle, but the smallest circle with the W-based collimators appears to be smaller due to a better resolution. The contrast levels with the hot-cased phantom with six hot circles were seen the same as with cold circles ( Fig. 4 ). As shown in Figs. 3 and 4 , Lu-177 provided better contrast for both cold and hot circles with LEHR W-based collimators than that for Pb, as with I-123. However, for Lu-177, there were little contrast differences with the ME collimators. The contrast for the hot-cased phantom with I-131 was comparable with ME W-based and HE Pb collimators.

## Discussion

This study investigated the potential positive effects of the system on functional parameters including spatial resolution, sensitivity, and image quality when using tungsten and tungsten alloys as collimator material of a gamma camera system. The study was conducted for planar imaging, but since SPECT is based on planar projection images, acquired around the patient, it is assumed that any improvement seen for planar images could translate to improvement in SPECT. Of course, there are steps in the reconstruction process that affect, for example, the spatial resolution such as noise regularization methods and number of iterations. On the other hand, SPECT with iterative reconstruction has a good possibility to compensate for the degradation of spatial resolution which is related to the events from collimator septal penetration if information of such events is included in the collimator response function (CRF) correction, that today are common in clinical software systems. It is, in principle, also possible to correct for penetration effects in planar images by creating filter functions that account for the addition of penetration to the PSF. Sjögreen et al have shown improvement in image quality in I-131 whole-body imaging by using a Wiener-like restauration method using Monte Carlo–based scatter kernels obtained from simulation of point sources at various depth in a water phantom. 31 This method has also been applied for ^90^ Y bremsstrahlung imaging. 32

For all cameras, there is a maximum weight that the gantry can carry. In this study, we simulated a camera system that can carry an HE Pb collimator of 126 kg. We did not include HE W-based collimators as, according to Table 2 , we assumed that these would be too heavy. The weights of the ME and LEHR W-based collimators were, based on our rescaling method, such that these could potentially be carried by the system. This implies that there are also possibilities to optimize the ME and LEHR collimators for better imaging properties than the current configurations based on Pb.

Current ME Pb collimator configurations are probably not optimal for Lu-177, which is an isotope that is increasingly used for therapy of neuroendocrine tumors with Lutetium Lu 177 dotatate that binds to somatostatin receptors on tumors and metastasized prostate cancer using a prostate-specific membrane antigen (PSMA) labeled to Lu-177. A reduced septal thickness is possible because of the better attenuation properties of W-based collimators, which could be reduced allowing for more holes with a related increase in sensitivity or spatial resolution. The use of a W-based collimator, optimized for 208 keV and spatial resolution, could improve image-based dosimetry procedures and lead to more accurate absorbed dose estimates compared with the present Pb-based ME collimators.

Our previous study on the image quality of Tc-99m and when using LE collimators of W and Wolfmet alloys showed the superiority of W alloy collimators compared with the Pb and Pb-Sb. 29 So the results are consistent with the findings in the present study for ME and HE collimators with I-131 and Lu-177 imaging. Finally, more studies with other hole geometries and energy ranges with the materials are needed for the optimal spatial resolution and septal penetration values.

## Conclusion

This study has shown that the use of potential collimators made of tungsten and Wolfmet tungsten alloy could provide an improved spatial resolution and image contrast by reducing septal penetration compared with conventional Pb-based collimators. The results indicate that the development of new collimators with better resolution as compared with the current HE and ME collimators could improve imaging with I-131 and Lu-177. A W-based LEHR collimator could improve the image quality for I-123 imaging and still be useful for Tc-99m imaging.
